# Quality of Life Assessment After Pelvic Prolapse Surgery With and Without Mesh: A Literature Review

**DOI:** 10.3390/jcm14041325

**Published:** 2025-02-17

**Authors:** Marilena Pirtea, Oana Balint, Cristina Secoșan, Dan Costăchescu, Alexandru Dabîca, Dan Navolan

**Affiliations:** 1 Doctoral School, University of Medicine and Pharmacy “Victor Babeș”, 300041 Timișoara, Romania; marilena.pirtea@umft.ro (M.P.); alexandru.dabica@umft.ro (A.D.); 2 Obstetrics-Gynecology I Department, University of Medicine and Pharmacy “Victor Babeș”, 300041 Timișoara, Romania; secosan.cristina@umft.ro (C.S.); navolan.dan@umft.ro (D.N.); 3 Radiology Department, University of Medicine and Pharmacy “Victor Babeș”, 300041 Timișoara, Romania; costachescu.dan@umft.ro

**Keywords:** pelvic organ prolapse, quality of life, mesh surgery, patient-reported outcomes

## Abstract

**Background/Objectives**: The quality of life (QoL) in patients undergoing pelvic prolapse surgery is a critical area of research in urogynecology. Pelvic organ prolapse (POP) is a prevalent condition affecting a significant number of women, leading to various physical and psychological symptoms that can severely impact their quality of life. Surgical intervention aims not only to correct the anatomical defects but also to enhance the overall well-being of patients. **Methods**: A comprehensive literature search in the main databases was conducted for studies evaluating quality of life after surgical treatment using techniques with and without mesh. **Results**: A total of 35 studies met the inclusion criteria, involving a total of 4603 patients. Twenty-two distinct patient-reported outcomes (PRO) questionnaires have been identified as post-surgical QoL assessment tools. **Conclusions**: Quality of life is significantly improved by surgical treatment of pelvic organ prolapse. Post-surgical PRO scores do not seem to be influenced by the surgical technique used, with no significant differences between methods using mesh or not.

## 1. Introduction

Pelvic organ prolapse (POP) is a condition in which the pelvic organs descend from their normal position and bulge into the vaginal canal due to weakened pelvic floor muscles and connective tissue. According to the most recent studies, the prevalence of the condition is approximately 28%. However, there is significant variability in the reported rates across literature due to the diverse diagnostic tools employed, the definition used, and the ethnicity of the population assessed [1]. Given its relatively high prevalence, POP and its management have a substantial impact on society and the healthcare system. Several risk factors have been identified, with vaginal delivery and the levator hiatal area being the most significant. However, a substantial association has also been reported with age, birthweight, and body mass index (BMI) [2]. POP can significantly impair physical, social, and psychological well-being. Patients often experience reduced physical activity, sexual dysfunction, and feelings of embarrassment or shame, which may lead to anxiety and depression. The severity of symptoms can affect personal relationships and daily functioning, leading to a diminished overall quality of life [3].

When conservative treatments such as pelvic floor exercises or pessary use do not provide adequate relief, surgery is the treatment of choice. Surgical techniques offer the advantage of reversing the gradual progression of pelvic organ prolapse (POP) and restoring anatomical support. Two primary surgical approaches for POP are currently employed in clinical practice: surgery involving synthetic mesh and surgery without the utilization of synthetic mesh. Surgical techniques employing mesh entail the implantation of synthetic mesh materials to augment the weakened pelvic floor structures, reinforcing vaginal walls, or supporting specific organs such as the bladder [4]. Mesh repairs have demonstrated a reduced recurrence rate compared to non-mesh repairs, but they are associated with a higher risk of complications, including mesh erosion, infection, and chronic pain. Consequently, the use of mesh has been subject to scrutiny in recent years, particularly in the context of vaginal mesh procedures [5]. Surgical techniques that avoid mesh use employ the patient’s own tissue to restore support and repair to the pelvic organs [4]. These non-mesh repairs rely on suturing the vaginal walls or repositioning the prolapsed organs. While these non-mesh repairs carry lower risks of complications, they tend to have a higher recurrence rate of prolapse over time compared to mesh-based repairs. This recurrence is particularly pronounced for more advanced grades of initial prolapse or in patients with significant vaginal laxity [6].

Quality of life (QoL) assessment following pelvic organ prolapse (POP) surgery is a critical component of evaluating treatment success. Surgical interventions aim to alleviate symptoms such as pelvic pressure, urinary incontinence, and sexual dysfunction, thereby improving overall quality of life. Patient-reported outcomes (PROs) are the gold standard for quality of life assessment post-surgery. Validated tools such as the Pelvic Floor Distress Inventory (PFDI) and Pelvic Floor Impact Questionnaire (PFIQ) are widely used to quantify symptom relief and functional recovery [7]. Studies consistently show that surgical repair improves both physical symptoms and mental well-being, particularly when associated with reduced anxiety, depression, and embarrassment caused by POP symptoms [4].

Additionally, sexual health, a key component of QoL, often improves post-surgery. Many women report reduced dyspareunia and improved sexual satisfaction, though outcomes can vary based on surgical technique and individual patient factors [8]. Despite these benefits, it is crucial to assess long-term outcomes, as prolapse recurrence and complications, such as mesh erosion or de novo urinary symptoms, can negatively affect QoL over time [9].

This literature review synthesizes findings from various studies to present current methods of QoL assessment and QoL outcomes between surgical techniques utilizing mesh and those employing native tissue repair methods.

## 2. Materials and Methods

The present review aims to present the existing evidence regarding the quality of life outcomes after pelvic organ prolapse surgery, both with and without synthetic mesh. The study adheres to the PRISMA statement.

- **Eligibility Criteria**

The inclusion criteria for study selection were as follows:

1. Study design: Original articles, including randomized trials and observational studies.
2. Method criteria: Studies that compare surgical techniques using mesh with surgical techniques not using mesh (sutures or native tissue repairs).
3. Outcomes reporting: Studies that assess quality of life after pelvic organ prolapse surgery.
4. Human involvement: Studies involving human participants.
5. Language: Studies published in English.
6. Full text availability: Studies accessible as complete text.

- **Information Sources and Search Strategy**

The search for eligible studies was conducted in medical databases such as PubMed, Scopus, and Web of Science. Studies published between January 2000 and November 2024 were considered. Additionally, references of identified relevant studies were manually searched for potential studies. The search strategy employed multiple association of keywords: “quality of life”, “mesh”, “without mesh”, “native tissue”, “pelvic organ prolapse”, “uterine prolapse”, “vaginal vault prolapse”, “cystocele”, and “rectocele”.

- **Study Selection**

After the initial search, 1386 articles were found. Their titles and abstracts were screened for eligibility, and articles that did not meet the inclusion criteria, were non-relevant or were duplicate studies were excluded. In the subsequent phase, the full texts of the remaining 74 studies were reviewed, and non-relevant articles were also eliminated. Thirty-five (n = 35) articles were ultimately included in the review.

- **Data Extraction**

Data relevant to the study objectives including first author, year of publication, design of the study, number of patients included, affected pelvic compartment, grade of prolapse (POP-Q classification), type of surgical intervention, time of assessment, type of QoL questionnaires used, QoL scores pre-surgery (baseline) and post-surgery, and QoL overall outcomes were extracted, and a database was created in Jamovi software ver. 2.6.24 (https://www.jamovi.org, accessed on 15 January 2025).

## 3. Results

**Figure 1:**
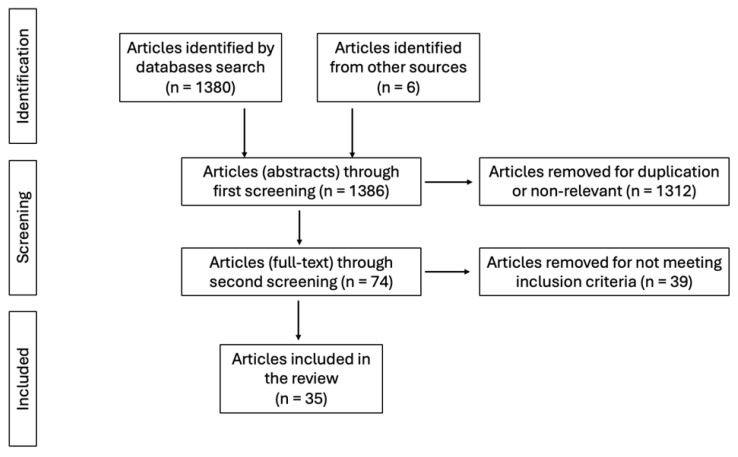
PRISMA Chart.

In total, 35 articles were considered eligible to be included in the review from an initial search of 1386 articles. The flowchart of the study is presented in Figure 1.

### 3.1. Characteristics of the Studies Included

Studies published between 2000 and 2024 were included in the analysis. All studies were designed as randomized controlled trials. 

### 3.2. Characteristics of the Population Included

The 35 eligible studies included a total of 4603 patients. Notably, the age of the patients was not a significant factor in the studies, as it did not constitute an inclusion or exclusion criterion, except for the requirement of being over 18 years of age. The included studies primarily focused on a single prolapsed compartment in 22 out of 35 studies. The anterior compartment was the most analyzed compartment, followed by the apical compartment, which encompassed either uterine prolapse or prolapse of the vaginal vault. Eight studies involved multiple compartments, with the anterior and posterior being the most frequently associated compartments. Additionally, five studies did not specify the compartment involved or included any compartment. The summary of the compartment analyzed is presented in Table 1. The grade of pelvic organ prolapse (POP) served as an inclusion criterion in 31 of the studies. Specifically, 25 studies utilized a POP-Q grade 2–4 for inclusion, while 6 studies included only severe stages of POP (POP-Q grade 3–4).

### 3.3. Surgical Techniques

This review analyzed studies that compared various surgical techniques for pelvic organ prolapse (POP) treatment, including those involving mesh placement and those not involving mesh, such as native tissue repairs or fixation techniques. The most prevalent association of surgical procedures examined was anterior colporrhaphy versus transvaginal mesh repair in eleven of the included studies (all involving anterior compartment prolapse). The procedures included vaginal, abdominal, and/or laparoscopic techniques. A summary of the surgical procedures utilized is presented in Table 1.

### 3.4. Time of Assessment

The assessment time varied across the included studies, ranging from three months to five years. Nineteen of the thirty-five studies reported their outcomes at twelve months.

### 3.5. Quality of Life Assessment Methods

Patient-reported outcomes (PROs) are essential in evaluating the impact of pelvic organ prolapse (POP) and its treatment on women’s quality of life. PROs capture patients’ perspectives on symptoms, daily functioning, and overall well-being, offering valuable insights beyond clinical measures. There are several validated PRO questionnaires used in POP to assess symptom severity and functional impairment [45]. Studies show that these instruments reliably measure improvements after POP surgery, highlighting the importance of patient-centered evaluations in guiding treatment decisions and assessing surgical success [7]. A summary of the PRO questionnaires used in the reviewed studies is presented in Table 2. The same questionnaires were used in the pre-surgery assessment for baseline scores and again at each time of assessment.

#### 3.5.1. Pelvic Floor Distress Inventory (PFDI)

The Pelvic Floor Distress Inventory (PFDI) is a condition-specific health-related quality of life (HRQOL) questionnaire designed by Barber et al. in 2001 to assess symptom distress in women with pelvic floor dysfunctions secondary to POP [46]. The PFDI includes three subscales: the Pelvic Organ Prolapse Distress Inventory (POPDI), the Colorectal-Anal Distress Inventory (CRADI), and the Urinary Distress Inventory (UDI). These subscales measure specific symptom domains, enabling a comprehensive understanding of the patient’s condition. In 2005, Barber concluded that although PFDI is a reliable tool, its comprehensive nature and length may limit its use in clinical and research purposes, and developed a short form, called PFDI-20 [7].

PFDI was the most frequently used method of quality-of-life assessment, utilized 47 times in 18 out of the 35 studies included. It was applied either in its short form, PFDI-20, or as its subscales, with the UDI-6 subscale being utilized in 15 of these instances.

#### 3.5.2. Pelvic Floor Impact Questionnaire (PFIQ)

The PFIQ is a HRQOL designed along with PFDI by Barber in his 2001 study. Its purpose is to assess the life impact in women with POP [46]. The PFIQ consists of three subscales: the Urinary Impact Questionnaire (UIQ), the Colorectal-Anal Impact Questionnaire (CRAIQ), and the Pelvic Organ Prolapse Impact Questionnaire (POPIQ). In 2005, a short form, PFIQ-7, was also developed for ease of use [7].

PFIQ use was identified in 14 of the 35 studies, with the second most used being PRO questionnaire (42 times), applied either in its short form, or as its subscales.

#### 3.5.3. The Pelvic Organ Prolapse/Urinary Incontinence Sexual Questionnaire

PFIQ is a validated and reliable 31-item questionnaire developed by Rogers in 2001 that evaluates sexual function in women with pelvic organ prolapse or urinary incontinence [47]. A short form, with 12 items (PFIQ-12), with a wider applicability was created by the same author two years later [48]. The use of PFIQ-12 is recommended with grade A evidence for sexual function assessment with urinary function by The International Continence Society [49]. The PISQ-12 is particularly valuable in evaluating treatment outcomes, such as surgical interventions or physical therapy, by capturing patient-reported changes in sexual function over time.

In the current review, PISQ-12 was used in 14 of the 35 studies.

#### 3.5.4. Prolapse Quality of Life Questionnaire

The P-QoL questionnaire was developed by Digesu in 2004 to address the limitations of existing PFDI and PFIQ questionnaires (which lacked a short form at the time) [50]. The questionnaire’s items were designed to highlight the impact of pelvic dysfunctions on the actual quality of life experienced by women. It comprises 38 questions distributed across eight domains of quality of life: general health, prolapse impact, role, physical and social limitations, personal relationships, emotional distress, sleep/energy disturbances, and their severity [51].

In our review, P-QoL was used in 7 of the 35 studies included.

#### 3.5.5. Other PROs

Besides the mentioned PROMs, another nine QoL questionnaires were identified in the reviewed studies: Female Sexual Function Index (FSFI), Overactive Bladder Questionnaire-8 (OAB-8), Quality of Sexual Function Scale (QSF), Patients Global Impression of Improvement (PGI-I), EQ-5D-5L, Incontinence Impact Questionnaire (IIQ-7), Defecatory Distress Inventory (DDI), Obstructed Defecation Syndrome Score (ODS), and King’s Health Questionnaire (KHQ). Their use was usually associated with the use of one of the main PROMs. In two studies, no specific questionnaire was used to assess post-surgical quality of life outcomes.

### 3.6. Quality of Life Outcomes After POP Surgery

Of the 35 studies included, which compared different surgical techniques with and without mesh, 28 studies reported a similar result, observing significant improvement in PROM scores compared with baseline (pre-surgery) with no significant differences in scores between study groups (with and without mesh) at time of assessment. A summary of the outcomes of each study is presented in Table 3. The mean pre-(baseline) and post-surgery values of the main PROs were analyzed, where available, and presented in Table 4. In 6 of the 35 studies, significant improvements in QoL were reported post-surgery with some mentions. Da Silveira observed that although the overall P-QoL scores were similar between groups, in six of the eight domains of the questionnaire, superior improvement was observed in the mesh group [42]. In the Dias study, significant improvement in both groups was reported with a similar post-operative P-QoL, but he also observed significantly improved patient satisfaction in the mesh group, probably due to the cofounders, such as simultaneous urinary incontinence treatment [12]. A higher EQ-5D-3L in the mesh kit group (vs. native tissue surgery) was reported by Glazener at 2 years assessment. No other significant differences were observed in other PROMs or satisfaction with treatment and neither in the EQ-5D-3L at 12 months [35]. A significant difference for UDI-6 scores, in favor of hysterectomy with uterosacral suspension (vs. sacrospinous hysteropexy) was reported by Nager. No other PROM scores were different between the two groups [40]. In Tamanini’s study, although no difference in scores was reported between groups at 60 months of assessment (anterior colporrhaphy vs. transvaginal mesh), a significant negative impact on quality of life was observed in the mesh group after adjusting for a series of cofounders [19]. In his 2020 RCT, van Ijsselmuiden compared laparoscopic sacro-histeropexy versus sacrospinous fixation. At 12 months, he observed significant improvements in QoL scores in both groups but significantly worse overactive bladder symptoms in UDI-6 score and incontinence symptoms in DDI scores for the laparoscopic hysteropexy group [25].

Only one study of da Silveira reported a significant difference in the PROM assessment between his groups (different procedures with native tissue fixation vs. hysterectomy with mesh fixation) with a better P-QoL score at 12 months for mesh group but with significant improvement in both groups [41]. This result was significant only for the anterior compartment prolapse; the groups with apical and posterior compartments did not show a statistically significant difference. The authors suggested that the discordance in their results as compared with existing literature may be due to the surgical technique used and the grade of prolapse (POP-Q III-IV).

Mesh exposure rate was reported in 24 of the 35 studies included and ranged between 2% and 35.7%, with most of the studies reporting under 15%. The highest rate, observed in Lopes’s study was justified by the author as due to the placing of the mesh over the rectovaginal fascia [30].

## 4. Discussion

In addition to anatomical restoration, pelvic organ prolapse (POP) surgery has specific post-surgical outcomes such as symptom severity, quality of life, and sexual function. This review presents a descriptive synthesis of quality of life assessment tools utilized after POP surgery. These specific tools encompass domains such as urinary, bowel, and sexual health. The selection of the most appropriate quality of life assessment tool for a study involves assessing the desired outcome, evaluating its sensitivity to changes in the outcome after surgery, aligning with the demographic and clinical characteristics of the study participants, and also adhering to recommendations from international professional associations or regulatory agencies.

Current POP management guidelines do not recommend a specific surgical procedure or surgical approach. Instead, tailoring surgical treatment to each patient’s circumstances is suggested [52,53]. The use of mesh techniques has been associated with a significantly reduced risk of objective recurrence compared with native tissue for anterior prolapse but with no difference for subjective outcomes [4]. Similar conclusions were reported for the middle compartment [54], while for the posterior compartment, no evidence recommends the use of mesh procedures [4]. However, despite the controversies surrounding the use of mesh procedures and no major benefits as compared with native tissue procedures, mesh surgery for POP remains the management of choice for many surgeons, especially surgeons with a high volume and those working in private practices [55].

This review included studies analyzing comparations of various surgical techniques restoring local anatomy using mesh with techniques restoring local anatomy using native tissue or fixations at different levels. The compartment affected and the grade of prolapse (POP-Q classification) varied among studies. The time of assessment had significant variability, with reports spanning from 6 months to 5 years.

The review included 35 studies that met the inclusion criteria and identified 22 distinct patient-reported outcome questionnaires (PROs) designed to evaluate various aspects of life quality. The most frequently utilized PROs were the Physical Functioning Disability Index (PFDI), specifically its short form (PFDI-20), and its other subscales. PFIQ, also in its short form, and its subscales were also commonly used. The quality of life assessed by the validated patient-reported outcome questionnaires has shown a substantial improvement after surgery in all the included studies, regardless of the compartment affected, the severity of prolapse, or the surgical technique employed. Notably, with a few exceptions, such as a specific scale or symptom of a certain PRO, there were no significant differences in quality of life between using mesh and not using it.

The mesh erosion ranged between 2% and 16%, with one exception of 35%, in accordance with the existing reports [56]. As mesh erosion is one of the most common complications of mesh surgery and one of the main reasons for it being controversial, the findings of this review show that quality of life does not seem to be significantly influenced by this complication. As mentioned earlier, the studies included had a mean time of assessment of 12 months (19/35 studies). The time from surgery to mesh exposure in pelvic organ prolapse (POP) repair varies across studies, with reports indicating a range from several weeks to a year postoperatively [57]. Thus, the chosen time of assessment for the included studies should reflect the impact of this mesh-specific complication on quality of life. However, the overall reintervention rates after mesh surgery are known to increase over time, with rates ranging from none at one year to 6.6% at two years and exceeding 20% at nine years [58,59,60]. While recurrence rates after native tissue surgery are reported as high as 42% at two years, further studies should be conducted to assess quality of life after longer periods of time in order to evaluate if the lack of difference between mesh or no mesh remains over time [4].

In recent years, a few other literature reviews focused on quality-of-life assessment after POP surgery. In 2022, Ghanbari et al. analyzed QoL outcomes after surgical interventions or pessary for POP. The review included different surgical techniques, vaginal reconstructive or obliterating, and abdominal open, laparoscopic or robotic. The author reported that almost all interventions were associated with an improvement in quality of life as assessed by using different PROs [61]. Similar findings were published by Guan, who analyzed different surgical techniques for POP but focused only on studies reporting its outcome using PFDI-20 and PFIQ-7 questionnaires [62].

The existing literature focusing on POP surgery outcomes predominantly presents a consistent finding that surgical treatment leads to improved quality of life, with minimal variation in outcomes among different techniques used. Currently, no ideal surgical approach has been identified, as each procedure entails certain disadvantages, such as a higher incidence of complications or recurrence. Guidelines recommend tailoring treatment to individual characteristics or informed preferences, which appears to be the most effective approach until further evidence or novel techniques emerge.

- **Strengths and limitations**

To the best of our knowledge, this is the first review that systematically evaluates the quality-of-life impact of randomized controlled trials (RCTs) comparing mesh techniques with native tissue techniques. The search strategy and assessment of the studies were conducted as standardized as possible, adhering to the methodology applied in other publications in the field.

The study’s limitations arise from the heterogeneity of the included studies regarding surgical methods, assessment time, and questionnaires utilized. These limitations are challenging to resolve, as currently, specific standards are lacking for these parameters.

## 5. Conclusions

Quality of life is significantly improved by surgical treatment of pelvic organ prolapse. Several patient-reported outcome (PRO) questionnaires have been validated for assessment of QoL in this group of patients, highlighting treatment success. Post-surgical PRO scores do not seem to be influenced by the surgical technique used, with no significant differences between methods using mesh or not.

## Figures and Tables

**Table 1:** Compartment and surgical techniques used in the included studies.

Compartment		Surgical Techniques	Studies
Single	Anterior	Anterior colporrhaphy vs. transvaginal mesh	[10,11,12,13,14,15,16,17,18,19,20]
		Vaginal colposuspension vs. transvaginal mesh	[21]
		Laparoscopic sacropexy vs. transvaginal mesh	[22]
		Abdominal paravaginal repair vs. anterior colporrhaphy with mesh	[23]
		Multiple native tissue techniques vs. transvaginal mesh	[24]
	Apical	Sacrospinous fixation vs. laparoscopic sacropexy	[25,26]
		Sacrospinous fixation vs. abdominal sacropexy	[27,28]
		Sacrospinous fixation vs. mesh;	[29,30]
		Uterosacral suspension vs. abdominal sacropexy	[31]
Multiple	Anterior/posterior	Native tissue techniques vs. transvaginal mesh	[32,33,34,35,36,37]
	Anterior/apical	Sacrospinous fixation vs. mesh;	[38]
		Uterosacral fixation vs. mesh;	[39]
	Any/Not specified	Uterosacral suspension vs. sacrospinous suspension with mesh;	[40]
		Multiple native tissue techniques vs. transvaginal mesh	[41,42,43,44]

**Table 2:** Summary of PRO questionnaires used in the included studies.

Study	PFDI				PFIQ				ICIQ			PISQ-12	FSFI	OAB-V8	P-QoL	QSF	PGI-I	EQ-5D-3L	IIQ-7	DDI	ODS	KHQ	Not Specified
	PFDI-20	UDI-6	POPDI-6	CRADI-8	PFIQ-7	UIQ-7	POPIQ-7	CRAIQ-7	ICIQ-VS	ICIQ-OAB	ICIQ-UI SF												
Allahdin [32]																							x
Carey [33]		x													x				x				
da Silveira [41]															x	x							
da Silveira [42]															x	x							
Daneshpajooh [26]	x				x							x											
de Tayrac [10]		x	x	x		x	x	x				x											
Delroy [11]															x								
Dias [12]															x								
Galad [38]																							
Glazener [34]									x									x					
Glazener [35]									x								x	x					
Gutman [43]	x	x	x	x	x	x	x	x				x											
Halaska [29]						x	x	x				x											
Iglesia [39]	x	x	x	x	x	x	x	x				x											
Juliato [27]									x	x	x												
Lamblin [21]	x	x	x	x	x	x	x	x															
Lopes [30]																						x	
Lucot [22]	x	x	x	x	x						x	x	x					x			x		
Madhuvrata [36]																							x
Menefee [13]		x	x			x	x					x											
Minassian [23]		x	x	x		x	x	x				x											
Nager [40]	x	x	x	x	x	x	x	x				x					x						
Nguyen [14]	x	x	x	x	x	x	x	x															
Rondini [31]	x											x			x								
Rudnicki [15]	x	x	x	x	x	x	x	x				x											
Rudnicki [16]	x				x							x											
Sivaslioglu [24]															x								
Sokol [44]	x	x	x	x	x	x	x	x				x											
Tamanini [17]											x			x								x	
Tamanini [18]									x		x			x									
Tamanini [19]									x		x			x									
van Ijsselmuiden [25]		x										x							x	x			
Vollebregt [20]		x																	x				
Withagen [37]		x															x		x	x			

x, PRO questionnaire used in the study.

**Table 3:** Characteristics of the studies included and summary of outcomes.

Study	Year	Design	Population	Outcome
Allahdin [32]	2008	RCT	66	Significant improvement with no difference between groups
Carey [33]	2009	RCT	139	Significant improvement with no difference between groups
da Silveira [41]	2019	RCT	122	No significant difference in domains of general health perception and personal relationship limits but significant improvement in all the other domains
da Silveira [42]	2014	RCT	184	Significant improvement in mesh group for anterior compartment only; no difference for apical and posterior compartments
Daneshpajooh [26]	2022	RCT	32	Improvement with no difference for all questionnaire scores
de Tayrac [10]	2013	RCT	147	Significant improvement with no difference between groups
Delroy [11]	2013	RCT	79	Significant improvement with no difference between groups
Dias [12]	2016	RCT	88	Significant improvement with better satisfaction in mesh group—probably by cofounders
Galad [38]	2020	RCT	146	Significant improvement with no difference between groups
Glazener [34]	2020	RCT	154	No difference between groups with the exception of EQ-5D-3L at 1 year in favor of mesh kit vs. native
Glazener [35]	2017	RCT	865	Significant improvement with no difference between groups
Gutman [43]	2013	RCT	65	Significant improvement with no difference between groups
Halaska [29]	2012	RCT	168	Significant improvement with no difference between groups
Iglesia [39]	2010	RCT	65	Significant improvement with no difference between groups
Juliato [27]	2018	RCT	71	Significant improvement with no difference between groups
Lamblin [21]	2014	RCT	78	Significant improvement with no difference between groups
Lopes [30]	2009	RCT	32	Significant improvement with no difference between groups
Lucot [22]	2021	RCT	262	Significant improvement with no difference between groups
Madhuvrata [36]	2011	RCT	66	Significant improvement with no difference between groups
Menefee [13]	2011	RCT	99	Significant improvement with no difference between groups
Minassian [23]	2014	RCT	70	Significant improvement with no difference between groups
Nager [40]	2021	RCT	118	Significant improvement with no difference between groups except for UDI with a significant improvement for HT group
Nguyen [14]	2008	RCT	76	Significant improvement with no difference between groups
Rondini [31]	2014	RCT	124	Significant improvement with no difference between groups
Rudnicki [15]	2013	RCT	161	Significant improvement with no difference between groups
Rudnicki [16]	2015	RCT	138	No difference between groups
Sivaslioglu [24]	2007	RCT	90	Significant improvement in both groups
Sokol [44]	2012	RCT	65	Significant improvement with no difference between groups
Tamanini [17]	2020	RCT	92	No significant difference; mesh group associated with negative impact after adjusting for other variables
Tamanini [18]	2013	RCT	100	Significant improvement in both groups
Tamanini [19]	2015	RCT	100	Significant improvement in both groups
van Ijsselmuiden [25]	2020	RCT	126	Significant improvement in both groups, OAB (from UDI) and fecal incontinence (from DDI) worse after LSH group
Vollebregt [20]	2011	RCT	125	Significant improvement with no difference between groups
Withagen [37]	2011	RCT	194	Significant improvement with no difference between groups

**Table 4:** Scores obtained on the most used PRO questionnaires in the included studies. No significant difference between mesh in no-mesh techniques.

PRO Questionnaire	Mean Preop. Score—Mesh	Mean Preop. Score—No Mesh	Mean Postop. Score—No Mesh	Mean Postop. Score—No Mesh
PFDI-20	95.6 (±28.5)	112 (±33.4)	32.7 (±11.8)	32.9 (±13.3)
			*p* = 0.97	
UDI-6	53.5 (±25.7)	56.1 (±27.1)	15.3 (±8.6)	14.1 (±6.7)
			*p* = 0.69	
POPDI-6	60.9 (±34.2)	66.7 (±28.5)	10 (±10.4)	13.4 (±11.6)
			*p* = 0.52	
CRADI-6	32.9 (±31.6)	42.9 (±33.2)	15.6 (±9.6)	18.6 (±15.1)
			*p* = 0.62	
PFIQ-7	42.3 (±23.4)	48.9 (±20.4)	9.05 (±8.6)	10.9 (±9.8)
			*p* = 0.65	
UIQ-7	29.7 (±24.2)	37.0 (±31.8)	10.8 (±10.8)	12.5 (±14.6)
			*p* = 0.78	
POPIQ-7	20.6 (±21.8)	27.2 (±25.8)	2.57 (±3.9)	5.31 (±7)
			*p* = 0.32	
CRAIQ-7	11.1 (±11.2)	18.1 (±24.6)	3.19 (±3.49)	8.97 (±15.4)
			*p* = 0.28	
PISQ-12	21.8 (±13.2)	21.8 (±13.3)	22.7 (±14.4)	22.9 (±14.4)
			*p* = 0.97	

## Data Availability

No new data were created.
